# Ancillary study management systems: a review of needs

**DOI:** 10.1186/1472-6947-13-5

**Published:** 2013-01-07

**Authors:** Elizabeth K Nelson, Britt Piehler, Adam Rauch, Sarah Ramsay, Drienna Holman, Smita Asare, Adam Asare, Mark Igra

**Affiliations:** 1 LabKey Software, Seattle, WA, USA; 2 Statistical Center for HIV/AIDS Research & Prevention (SCHARP), Fred Hutchinson Cancer Research Center, Seattle, WA, USA; 3 University of California, San Francisco, CA, USA; 4 Immune Tolerance Network, Bethesda, MD, USA

## Abstract

**Background:**

The valuable clinical data, specimens, and assay results collected during a primary clinical trial or observational study can enable researchers to answer additional, pressing questions with relatively small investments in new measurements. However, management of such follow-on, “ancillary” studies is complex. It requires coordinating across institutions, sites, repositories, and approval boards, as well as distributing, integrating, and analyzing diverse data types. General-purpose software systems that simplify the management of ancillary studies have not yet been explored in the research literature.

**Methods:**

We have identified requirements for ancillary study management primarily as part of our ongoing work with a number of large research consortia. These organizations include the Center for HIV/AIDS Vaccine Immunology (CHAVI), the Immune Tolerance Network (ITN), the HIV Vaccine Trials Network (HVTN), the U.S. Military HIV Research Program (MHRP), and the Network for Pancreatic Organ Donors with Diabetes (nPOD). We also consulted with researchers at a range of other disease research organizations regarding their workflows and data management strategies. Lastly, to enhance breadth, we reviewed process documents for ancillary study management from other organizations.

**Results:**

By exploring characteristics of ancillary studies, we identify differentiating requirements and scenarios for ancillary study management systems (ASMSs). Distinguishing characteristics of ancillary studies may include the collection of additional measurements (particularly new analyses of existing specimens); the initiation of studies by investigators unaffiliated with the original study; cross-protocol data pooling and analysis; pre-existing participant consent; and pre-existing data context and provenance. For an ASMS to address these characteristics, it would need to address both operational requirements (*e.g.*, allocating existing specimens) and data management requirements (*e.g.*, securely distributing and integrating primary and ancillary data).

**Conclusions:**

The scenarios and requirements we describe can help guide the development of systems that make conducting ancillary studies easier, less expensive, and less error-prone. Given the relatively consistent characteristics and challenges of ancillary study management, general-purpose ASMSs are likely to be useful to a wide range of organizations. Using the requirements identified in this paper, we are currently developing an open-source, general-purpose ASMS based on LabKey Server (http://www.labkey.org) in collaboration with CHAVI, the ITN and nPOD.

## Background

Ancillary studies allow researchers to leverage the high-value data collected as part of a primary clinical trial or observational study, augment this data with additional measurements, and answer questions that were not part of the primary study design [1]. For example, an ancillary study might identify the characteristics of individual immune responses or viral types that contribute to vaccine failure or success, elucidate mechanisms of treatment response, or identify biomarkers associated with positive outcomes. Results from such studies can advance translational research and point the way to better treatments and trials. Ancillary studies can provide these benefits in a cost-effective manner because the bulk of study data has already been collected and, typically, the primary study has already shown a result worthy of further investigation.

The National Institute of Health (NIH) considers studies that reuse clinical trial or observational study data to be sufficiently desirable and common that it provides numerous, ongoing grant mechanisms [2-9] for funding them. Searching PubMed for the term “ancillary study” and its plural produces 910 results ([10], December 2012). A recent review of the emerging field of clinical research informatics identifies secondary data use as a key pain point [11,12]. From a broader perspective, reuse of scientific data has become a priority and a concern across fields of all science, not just biomedical research studies [13,14].

For primary studies and trials, clinical trial management systems (CTMSs) are widely used to enhance efficiency, reduce costs, comply with regulations, and speed up data analysis. Today, researchers have a wide range of CTMS options at their disposal, from proprietary solutions such as Oracle Corporation’s Phase Forward Clintrial [15] and Oracle Clinical [16] and to open source solutions such as TrialDB [17] and OpenClinica [18,19]. CTMSs are actively studied and developed by the academic community [20-24].

In contrast, with the exception of our own conference abstract [25], the research literature contains little discussion of systems (or system extensions) that specifically address the cradle-to-grave needs of ancillary studies. The open source i2b2 system (Informatics for Integrating Biology and the Bedside) [26-28] is particularly noteworthy for its support for de-identified cohort discovery across federated patient information repositories. However, descriptions of i2b2 focus primarily on the repurposing of information and material by-products of health care delivery [26-29], not on the full set of scenarios surrounding reuse of the products of trials and studies.

Given the importance, cost-effectiveness and prevalence of ancillary studies, as well as the wide use of CTMSs, it is surprising that general-purpose ancillary study management systems (ASMSs) have not been discussed in the research literature, either as stand-alone systems or as extensions to CTMSs. Just like CTMSs, ASMSs have the potential to ease the cost, administrative burden, and expertise required to execute ancillary studies. Given this potential, here we review the core scenarios that a general-purpose ASMS should support to provide the greatest benefits. We focus specifically on differentiating requirements for managing ancillary study data (*vs.* primary study data) to illustrate how the requirements for an ASMS extend beyond those of primary study management systems.

Our analysis is based upon our ongoing work to build an open-source, general-purpose ASMS based on LabKey Server [30-33] in collaboration with thee large disease research networks: (i) the Center for HIV/AIDS Vaccine Immunology (CHAVI) [34], (ii) the Immune Tolerance Network (ITN) [35,36], and (iii) the Juvenile Diabetes Research Foundation (JDRF) Network for Pancreatic Organ Donors with Diabetes (nPOD) [37-39]. General-purpose ASMS features are being built into the LabKey Server platform, so they are available in all updated instances of the system. Installations of LabKey Server that currently support our ASMS collaborators include the Atlas Science Portal and ITN TrialShare. The Atlas Science Portal [30,40] is customized and maintained by the Statistical Center for HIV/AIDS Research and Prevention (SCHARP) at the Fred Hutchinson Cancer Research Center (FHCRC) to support multiple HIV research networks, including CHAVI. ITN TrialShare [41] is customized and maintained by the ITN to support data sharing and collaboration among immunology researchers within and beyond the ITN. nPOD is currently setting up its own LabKey Server to support ancillary studies performed on its repository of high-value specimens from Type 1 diabetes donors.

Our investigation also benefited from assisting the HIV Vaccine Trials Network (HVTN) [42] and the U.S. Military HIV Research Program (MHRP) [43] in their efforts to develop custom, organization-specific tools for managing the operations of ancillary studies. These tools are built into the Atlas Science Portal.

Examples of ancillary studies currently being conducted by our collaborators include work to discover transplant rejection biomarkers (*e.g.*, genomic studies of liver allograft rejection and recurrent hepatitis C disease [44]), identify and investigate individuals with exceptional responses to infection (*e.g.,* CHAVI’s studies of “elite controllers” of HIV [45]); examine whether HIV vaccines exert selection pressure on viral sequences (*e.g*., the HVTN’s studies of vaccine recipients who became infected in the STEP trial [46]); and explore potential correlates of protection in HIV vaccine recipients (*e.g.*, the HVTN’s investigations of the STEP trial and the MHRP’s work on the Thai Phase III trial, also known as RV144 [47]).

## Methods

We identified requirements for ancillary study management primarily as part of our ongoing work with CHAVI, ITN, nPOD, HVTN, MHRP, and SCHARP. SCHARP is the data management center for CHAVI, HVTN, the Collaboration for AIDS Vaccine Discovery (CAVD), the HIV Prevention Trials Network (HPTN), and the Microbicide Trials Network (MTN). This work includes developing tools for HVTN and MHRP to facilitate operational management of the RV144 immune correlates study, a large-scale ancillary study that includes over 45 sub-studies.

We also consulted with researchers at a variety of disease research organizations regarding their workflows and data management strategies, including: CHAVI, ITN, nPOD, HVTN, SCHARP, MTN, HPTN, CAVD, the Women’s Health Initiative (WHI), and the Pacific Northwest Prostate Cancer Specialized Program of Research Excellence (SPORE). Lastly, we reviewed process documents for ancillary study management from a variety of other organizations to increase the breadth of our review [48-57].

The organizations we consulted manage large numbers of trials and studies all over the globe. For example, as of August 2011, the ITN had 42 active primary trials and numerous ancillary trials taking place at 158 domestic and 10 international sites. CHAVI had 5 active primary studies and several ancillary studies running at 14 sites worldwide. The HVTN had over 54 active primary studies and several large-scale ancillary studies taking place at over 40 sites in the U.S. and internationally. The HPTN had 18 active primary studies and 5 active ancillary studies taking place at over 45 sites worldwide. As of August 2012, nPOD reported that 82 ancillary studies at 59 different universities were using specimens from its repository [58].

The process we used to define and verify ancillary study management scenarios and requirements was deliberatively iterative. We conducted repeated interviews with collaborators working with representative organizations, particularly CHAVI, ITN, HVTN, MHRP, SCHARP, HPTN and MTN. We provided written copies of the scenarios and requirements distilled from these interviews to interviewees at these organizations. After individuals reviewed these written materials, we conducted follow-up interviews and updated the written scenarios and requirements to reflect detailed feedback. The ITN, HVTN, MHRP and SCHARP collaborators participating as co-authors provided written sign-off on the final set of scenarios and requirements. We are concretely validating these scenarios and requirements as we develop the ancillary study management features of LabKey Server and verify these features with researchers using the system to conduct ancillary studies.

## Results

### Differentiating characteristics of ancillary studies

Several qualities generally distinguish ancillary studies from typical clinical trials or studies of other types. These characteristics produce different core requirements for an ASMS than for a primary study management system. Some of these characteristics may exist for both types of systems; however, taken together, they are not typical for primary studies, so their associated requirements are not ordinarily satisfied by existing systems.

#### A. Collection of additional measurements

“Ancillary studies” are most commonly defined as secondary or exploratory studies that were not included in the primary study plan and require the collection of additional measurements [1]. The term “ancillary studies” may sometimes be broadened to encompass “secondary data analysis” [59]. Secondary data analysis does not involve further measurements [1], just the reanalysis of existing data. Colloquially, many terms may refer to ancillary studies, including “freezer studies” (because frozen specimens are used), “sub-studies” and “special emphasis studies.”

For our collaborators and the groups we consulted, collection of additional measurements typically means performing additional analyses on existing specimens from a primary study. Using existing, frozen specimens poses thorny challenges in locating, confirming consent, allocating, and transporting specimens to a range of labs for a multiplicity of assays. For example, as of August 2011, MHRP’s ongoing RV144 immune correlates ancillary study already involves over 100 different assays being performed at more than 40 separate labs.

For certain organizations, the collection of additional data as part of an ancillary study may also involve further clinical visits, clinical procedures, and/or collection of specimens. However, when a study requires new visits, procedure or specimens, it starts to share many characteristics (and requirements) of a primary study. For this reason, we focus here on ancillary studies that involve simply the analysis of frozen, existing specimens. This type of ancillary “freezer” study has informatics requirements most clearly different than a primary study and, in our experience, is the most common type of ancillary study.

#### B. Conduct of a study by external investigators

Depending on the organization, ancillary studies may be proposed by investigators who participated in the original study, or by researchers fully external to the project. After approval, studies may be run independently by external investigators, internally by the primary study organization, or collaboratively by a mix of both.

Management of data and operations is more complex when an external team runs all or parts of the ancillary study. Internal investigators can leverage the original study’s staff and systems possibly while the original study is ongoing. In contrast, external investigators face process- and technology-based barriers to getting primary study information and specimens; correctly interpreting primary data; ensuring data security; and contributing their results back to the primary study repository.

####  C. Pre-existing data context and provenance

Tracking and retention of data context (*e.g*., participant enrollment criteria, study location, *etc.*) and provenance (*e.g.*, which piece of data came from which source or study, which quality control or processing steps have taken place, *etc.*) can be challenging for all kinds of studies. If context and provenance information for a primary study are inaccessible, it may be impossible to reuse the study’s data correctly.

#### D. Cross-protocol data pooling and analysis

Ancillary studies often involve the comparison of cohorts who experienced different treatment protocols in separate primary trials, or pooling of subsets of participants across different primary studies for use in new analyses. Both of these techniques require tagging groups of participants across studies, and aligning and integrating data for these participants across protocols.

In order to integrate data across protocols, it may be necessary to reconcile differences in data representation across studies. For example, different laboratories may have used different controlled terminologies to record results in different studies. Context and provenance information are also needed to judge and/or enhance the comparability of pooled data.

Cross-study data reconciliation and integration can be even more challenging when each relevant primary study has been performed by a different organization, each with its own data management system and standards for data representation.

#### E. Pre-existing and new participant consent

Participants in the original trial may or may not have provided their consent for inclusion of their data and specimens in an ancillary study. Even when they have consented to reuse of certain types of data and specimens collected from them, they may not have consented to the reuse of all types for all purposes. For example, genetic analysis may be prohibited or not specifically included in the consent. This means that study managers may need to track down existing consent forms (and any revisions) to determine whether consent is sufficient, or whether further consent is needed.

### Existing alternatives for managing ancillary studies

Today, three strategies for ancillary study management seem to be most commonly employed: (i) using a CTMS for the aspects of ancillary studies that the tool can support (ii) using an *ad hoc* combination of software for project management, plus a second, separate tool for data management and a third tool for specimen requests and tracking or (iii) developing of custom, organization-specific, end-to-end systems. These three strategies are discussed here due to the frequency of their use, but they are not necessarily comparable, particularly for real-time study management for multicenter projects. For example, an *ad hoc* solution is unlikely to achieve the same level of data safety, reliability, consistency and management efficiency as a centralized, web-based information system.

#### i. CTMSs

A CTMS that successfully manages a primary clinical trial or observational study may not be well-suited to support ancillary study management. First and foremost, a CTMS may not be designed to support cross-study data pooling. Few existing CTMSs mention cross-protocol or cross-study analysis in their documentation. Furthermore, certain ancillary study data management scenarios (such as populating an ancillary study with primary data) are not typically required for the completion of a primary clinical trial or study, so they may not be supported. In direct opposition to ancillary study investigators’ need to accumulate new data, Good Clinical Data Management Practice (GCDMP) calls for the clinical trial database to be locked upon completion of the trial [60,61], so data cannot easily be added. Also, a CTMS may not be designed to manage or provide data access to external investigators who wish to initiate ancillary studies.

#### iia. Project management tools

Software such as Excel, Microsoft Project, Microsoft SharePoint, email and FTP can be used to support the operational side of ancillary study management. However, employing a variety of tools in concert can be more complex than using an end-to-end solution that spans common scenarios for ancillary study management. Many (though not all) project management tools lack support for multi-user collaboration. Furthermore, some tools do not “scale up” well. For example, managing projects in Excel is straightforward for small projects, but harder for large studies with thousands of subjects, multiple project managers and many participating institutions.

#### iib. Data management tools

In our review, SAS was mentioned most often as the tool used for managing and analyzing data in ancillary studies; however, extracting data with SAS requires programming expertise. STATA, SPSS and R were also mentioned [62], but these also require programming expertise. Excel and Access do not require extensive programming expertise; however, they do not inherently understand study concepts (such as visits, participants, and cohorts) or relationships between these concepts. Furthermore, these tools can pose challenges in scalability, multi-user access, and security.

We have previously reviewed open source systems for managing and integrating clinical and experimental data types; however, none of these platforms addresses the particular needs of ancillary studies [30]. Pietrobon *et al.* described a suite of tools for secondary analysis of study data, but these are intended for use in a manner fully external to the original study; furthermore, they focus on publicly available data [63].

Proprietary software systems for data integration (*e.g*., Oracle Life Sciences Data Hub [64] and SAS Clinical Data Integration [65]) certainly exist. The Oracle and SAS offerings are notable for their emphasis on cross-study data integration scenarios. However, proprietary systems have a number of limitations, including vendor lock-in, lack of transparency, limited extensibility, and cost, as we have reviewed previously [30].

#### iic. Specimen request tracking tools

Specialized tools, such as BSI (Biological Specimen Inventory System) [66] or Freezerworks [67], can be used for specimen management; however, they may not integrate smoothly with other systems involved in data and specimen management.

#### iii. Custom systems

Organization-specific solutions may range from integrated suites of common tools (*e.g.*, SAS datasets and programs used in conjunction with DataFax) to single-purpose systems built from the ground up to meet an organization’s particular needs. Such solutions can be completely satisfactory for ancillary studies, but may require programming expertise (often SAS), or entail high development costs.

Some proprietary systems (*e.g.*, Labmatrix from BioFortis [68]) provide support for certain ancillary study scenarios (particularly the exploration of existing data and specimens). However, it is difficult to evaluate how well such systems support the full operational and data management workflows of ancillary studies given the limited documentation publicly available for them.

### Key scenarios for ancillary study management

The steps described in this section provide a representative (but certainly not universal) workflow for ancillary study management. To focus the discussion, we consider a “freezer study” workflow, where existing specimens undergo additional analysis, but no new specimens or clinical data are collected. Our collaborators are most commonly concerned with this kind of ancillary study. This type of ancillary study is particularly illustrative of how the requirements for managing ancillary studies extend beyond those of either primary studies or secondary data analysis.

**Figure 1:**
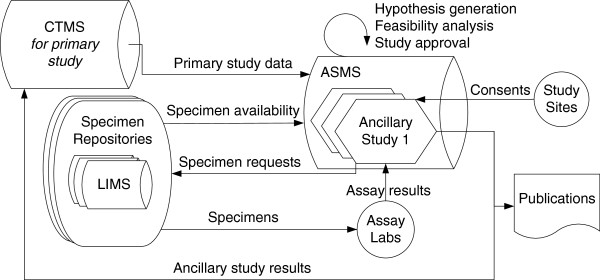
**ASMS data flows.** This figure shows a conceptual model for data flows for ancillary studies whose primary focus is analysis of stored specimens. In this scenario, data flows into the ASMS from the primary study’s CTMS (which contains information on participants, visits, consent and other pre-existing data) and specimen repository LIMSs (Laboratory Information Management Systems, which contain information on stored specimens available for further investigation). Before an ancillary study is initiated, the ASMS is used for hypothesis generation and feasibility investigations based on specimen availability. Once a particular ancillary study has been identified, a container for its data is established within the ASMS. After the ancillary study has been approved, any additional participant consents required for the study are collected by clinical sites and noted in the ASMS. Requests for needed specimens (including material transfer agreements) are sent to the appropriate specimen repositories, which in turn send stored specimens to appropriate labs. The labs perform assays on the specimens and import the results to the ancillary study container in the ASMS. Once the ancillary study is complete, results may be repatriated to the primary study. Results may also be shared in publications or other venues. This model presumes that all data for the ancillary study is managed within the ASMS, not the CTMS or an external system. It also presumes that external investigators can be given access to the study within the ASMS. Under different assumptions, usage patterns and data flows would change, but an ASMS could still prove helpful. For example, if gathering new clinical data from study participants is a significant piece of an ancillary study, using an organization’s existing CTMS for collecting and managing clinical data might make the most sense. An ASMS could still be desirable for other aspects of the study. For our collaborators, CTMSs have not proven amenable to the kinds of queries necessary for hypothesis generation and participant identification. Also, they are not ordinarily well-integrated with relevant LIMSs, so they do not facilitate identification of specimen availability. An ASMS could be used for these steps and others that are not typically supported by CTMSs or LIMSs, such as specimen requests and assay data management.

**Figure 2:**
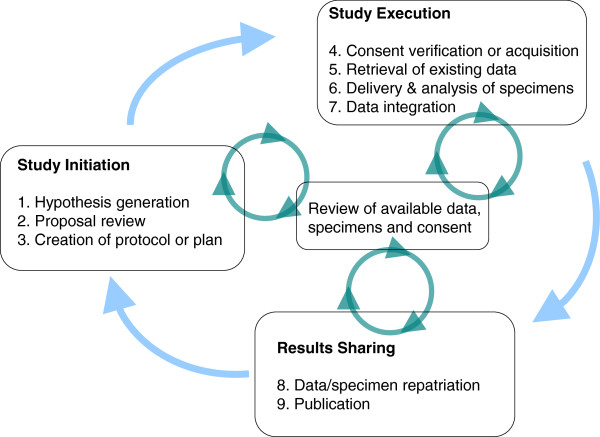
**Virtuous cycle feedback loops.** Using existing results and materials to refine hypotheses and develop new insights can produce “virtuous cycles,” where the research efforts of today feed tomorrow’s discoveries. Figure 2 shows two kinds of such cycles that are implied by the ancillary study workflow described here. An ASMS can facilitate both types of cycles by smoothing the flow of information, enabling collaboration, simplifying workflows and allowing researchers to make the most of existing materials and information. **(i) Full study cycle.** The nine steps in the ancillary study workflow form a virtuous cycle that spans the full life of a study, from the first glimmer of an idea through publication. For simplicity, Figure 2 breaks these steps into three phases (study initiation, study execution, and results sharing). These steps are roughly equivalent to those that form the “inner,” study-based loop in Kahn and Weng’s conceptual model for clinical research informatics [96]. In such cycles, published hypotheses and shared data from completed studies are used to generate future discovery cycles by providing inspiration and ingredients for follow-up studies. **(2) Incremental review cycles.** An ASMS can also facilitate smaller-scale virtuous cycles during all phases of an ancillary study. First and foremost, during the study initiation phase, the information and tools made available by an ASMS allow incremental refinement of hypotheses and study plans according to existing data, specimen availability, and consent limitations. During later phases of a study, an ASMS can make it easier to share and review new information as it is collected, allowing feedback of new insights into study investigations, operations, analyses, and conclusions. Of course, in-progress studies governed by clinical trial regulations will provide less scope for immediate use of this type of feedback than the kinds of pre-clinical, exploratory studies common among our collaborators.

For each step in this workflow, we describe scenarios where the support of an ASMS would be particularly valuable. Table 1 provides an overview of the steps and highlights the scenarios and requirements that most differentiate the needs of an ancillary study from those of a primary study. Figure 1 shows how data might flow to/from an ASMS for this workflow. Figure 2 shows two kinds of feedback loops (“virtuous circles”) implied by this workflow that could be facilitated by an ASMS.

**Table 1:** Differentiating requirements for a representative ancillary study workflow

***Step #***	***Step Name***	***Key Differentiating Requirements***
**1**	**Hypothesis generation**	a. Identify interesting categories of participants from primary study
		b. Access existing data and specimen information for those participants
		c. Provide sufficient information to external investigators for them to propose ancillary studies
**2**	**Proposal review**	a. Review availability of existing specimens
		b. Review priorities for use of remaining specimens, possibly reserving specimens
		c. Evaluate overlap and comparability of existing data and proposed measurements/analyses
**3**	**Creation of protocol or plan**	a. Decide which existing participant data and specimens to use.
		b. Plan expectations for collecting new ancillary data complementary to existing primary data
**4**	**Consent verification or acquisition**	a. Determine whether consent exists and is sufficient for desired analyses
		b. Obtain additional consent if necessary
**5**	**Retrieval of existing data**	a. Compile relevant subset of primary study data required for the ancillary study
		b. If external investigators are leading the ancillary study, share this subset of data with them.
**6**	**Delivery and analysis of specimens**	a. Request, locate, ship and track existing specimens, as well as manage material transfer agreements
		b. Maintain identifiers relevant to primary study during further specimen analysis
**7**	**Data integration**	a. Retain context and provenance from both primary and ancillary studies, including processing and quality control information
		b. Retain origination information (primary *vs.* ancillary)
		c. Resolve differences (representation, quality control, *etc.*) and join ancillary and primary data
**8**	**Data/specimen repatriation**	a. Contribute ancillary study data (raw and/or processed) back to the primary study
		b. Retain data context, provenance, processing and other metadata from ancillary study
		c. Return unused specimens
**9**	**Publication**	a. Coordinate preparation and review of publications across primary and ancillary investigators

This table calls out the key differentiating requirements for each step in the ancillary study workflow described in this paper. It aims to clarify how ancillary study requirements extend beyond those of primary studies and secondary data analysis. Note that this workflow addresses the “freezer study” scenario, where the ancillary study’s additional measurements come from analysis of existing specimens, not collection of new specimens or clinical data.

Any organization executing these kinds of ancillary studies would have its own unique workflow, using these steps to varying degrees and in varying order, so an ASMS would need to be sufficiently flexible to accommodate this variability. Nevertheless, our review revealed surprising consistency in scenarios, likely due to the uniformity of regulatory funding requirements and published best practices.

An ASMS could support only some of the steps in this workflow and still provide value. We heard repeatedly that disease research organizations want tools that can complement and seamlessly integrate with existing systems that already support certain scenarios, not replace all existing infrastructure. Workflow requirements that differentiate ancillary study management (highlighted in Table 1) are least likely to be covered well by existing tools.

In addition to facilitating the individual steps in this workflow, an ASMS might also facilitate overall project management for organizations facing a proliferation of ancillary studies. For example, an ASMS might track status and metadata for all of an organization’s ancillary studies; provide live summary dashboards for progress, materials and issues; and deliver notification emails upon completion of key steps.

#### 1. Hypothesis generation

This may take place at any point from initiation of the primary study onwards. It requires access to data and data context information, plus tools for making sense of information that may come from multiple studies.

The more broadly primary study data and context information can be made available in an accessible form, the broader the pool of investigators that can bring ideas to bear. Providing secure access to primary study datasets, analysis tools and curated materials (*e.g.,* related manuscripts) could attract potential investigators to areas of interest to the sponsoring organization. The greater an investigator’s access to such information, the more insightfully the researcher will be able to formulate hypotheses.

When exploring a potential hypothesis, when evaluating a proposal’s feasibility (Step 2), or when an ancillary study proposal and/or protocol has been accepted, relevant subjects, specimens and data must be identified across a range of data sources, often including multiple primary studies. To review participants for inclusion in an ancillary study, investigators need to align, integrate and evaluate a range of data types, including consent, clinical, assay, and specimen. To evaluate which types of data are comparable across studies, investigators need to access context and provenance information from the primary study. They also need to determine the availability, quantities, and quality of relevant specimens. The deeper the investigation possible at this stage, the less likely that a study will later be proven infeasible due to a lack of available participants meeting protocol requirements, or missing data, specimens, or consent.

To identify relevant participants and materials, researchers and administrators need tools for applying set theory relationships (*e.g.*, set difference, intersection, union, complement, *etc.*) and aligning, filtering, sorting, binning, combining, and visualizing diverse data types in a way that facilitates comparisons between participants. Ideally, study administrators should be able to select participants and datasets from multiple studies, align measurements across datasets and investigate these datasets without programming. Data reviewers also need a tagging system to keep track of participant groupings and designations, such as participants with relevant characteristics, for inclusion in study cohorts.

#### 2. Proposal review

In this step, an investigator submits a proposal that complies with the specific guidelines of the organizations sponsoring and conducting the primary study. This proposal typically describes the goals and relevance of the study; schedule expectations; general profiles of participants desired for the study; types of clinical data needed; labs or sites that expect to participate in the study; whether new clinical data or specimens are needed; whether existing specimens are required; the minimum specimen quantities necessary for analysis; plans for additional assays on specimens; any burden of analytical support that might fall on the original study’s data management and statistics team; and funding sources for external investigators and any internal support they require.

Proposals are vetted by the leadership of the original study (including specimen managers and statisticians), a range of review boards (*e.g.*, institutional review boards (IRBs)), funders, federal regulatory or sponsoring agencies, and participating clinic sites. If changes in a proposal are needed later (*e.g*., additional participant data are required), further review by these bodies may be necessary.

To perform satisfactory reviews of proposals, the team that sponsored the original study may need to review the data and unused specimens from that study, plus any competing requests for these specimens. That means that this information needs to be accessible, reviewable, and shareable in a secure manner. Tools that support data and specimen investigation and review (described in Step 1) may also be valuable here.

The breadth, diversity, and ongoing nature of review for ancillary study proposals mean that the review process can pose a significant operational load. For this reason, an ASMS should provide tools for submitting, requesting, tracking, and delivering materials, plus tracking the approval process and determining the status of applications.

#### 3. Creation of protocol or plan

The trial protocol or observational study plan sets out the research hypothesis, existing specimens, and new assays that are required, as well as data analysis and delivery expectations. Sometimes this document must be included in the original proposal that is reviewed in Step 2. However, earlier development of a complete protocol may not be possible when an external investigator lacks access to the data, context, specimens, and statisticians associated with the original study. Tools for providing limited access earlier in this process could reduce later iteration on proposals and protocols.

No matter when a protocol or proposal is developed, it will need to be tracked and made available to people involved in both new and original studies, ideally on a secure, restricted web site. It may also need to undergo further formal review and approval, so Step 2 may be repeated.

If analysis of existing specimens is needed, statisticians or scientists associated with the original study may provide investigation and guidance on which sets of samples to use. Communication of information between internal and external contributors at this stage might be facilitated by a secure, mutually accessible ASMS.

Tool support from an ASMS would also aid study design and expectation tracking. Both study design and expectation tracking for primary studies are commonly supported by CTMSs, but these CTMSs may not fully support cross-study planning. A study design tool allows generation of study plans (*e.g.*, visit/subject/assay mappings and expectations) from study parameters. Expectation tracking tools facilitate scheduling and tracking steps in a study (*e.g.,* which assay should be performed when and by which lab). This makes it easier to identify missed steps and to forecast schedule risk. Expectation tracking tools are particularly important when an ancillary study starts mid-way through a primary study and relies upon data or specimens that will be collected later in the primary study.

#### 4. Consent verification or acquisition

In order to use data or specimens from human subjects who participated in a primary study, investigators must either confirm consent using pre-existing consent forms or obtain new consent from participants. Central archiving of consent forms and/or consent metadata may facilitate determination of consent, but centralization may not have happened as part of the primary study.

Electronic management of consent is highly desired for all types of studies, not just ancillary studies [69], as are ontology-based definition of consent [70,71] and collection of metadata about consent coverage. These enable standardization of consent across network trials; reduce ambiguity; allow more efficient and consistent determination of consent coverage and status; increase the likelihood that specimens and data can be re-used in future studies; and facilitate compliance with consent agreements that require the destruction of participant specimens at a particular time or upon participant withdrawal from any phase of a trial.

#### 5. Retrieval of existing data

Once participants are identified and consent is confirmed, existing participant data records from relevant primary studies must be retrieved for reuse. Reconciliation of vocabulary (particularly column, field, visit, and participant identifier naming) and/or data representations (such as data types, units and formats) may be necessary to avoid conflicts and allow eventual integration. Tools that automate renaming, aliasing, and/or field parsing can help facilitate this process. Standardization may be required for all kinds of data, particularly when there is variation in study protocols, participant visit schedules, lab instruments, reagents, lab protocols or other aspects of data collection. Alignment of events or visits across studies or datasets is usually required for comparisons, but can be challenging. Both standardization and alignment typically require the assistance of expert statisticians. Statisticians’ work can be facilitated by tools that make it easier to draw data from multiple sources; determine data provenance and context; map visits; and filter, sort, align, integrate, and visualize the data.

Once data are retrieved, they need to be transmitted to ancillary study investigators, or external investigators need to receive access to the data on the sponsoring organization’s data systems. Today, when data are transmitted externally, they are often “thrown over the wall” to external investigators via SAS, Excel or text files. Data delivery may happen via email, which can pose security concerns. The external investigator then uses Excel, Access, SAS, or other software to accumulate and analyze results, sometimes completely independently. When primary data are delivered in a one-time snapshot, corrections to data in the primary study may not be passed on. Furthermore, the arms-length strategy can produce downstream challenges when the sponsoring organization wishes to integrate data and results from an ancillary study into the primary, central database. The arms-length strategy also breaks links to related and/or contextual information, as well as pre-empting any automatic tracking of data provenance.

Alternatively, it might make more sense to provide external investigators with partial access to the sponsoring organization’s ASMS. Data formats and standards would have fewer opportunities to diverge if external investigators could use the ASMS’s tools, standards, and formats for managing data records, context, and provenance. An ASMS could also facilitate management of data transfer agreements, enforcement of data security restrictions, and monitoring of external data access.

#### 6. Delivery and analysis of specimens

Samples sufficient for performing the assays required for the study must be located, requested and shipped to appropriate labs in the right quantities at the right time for analysis. Furthermore, as part of the specimen request process, Material Transfer Agreements (MTAs) must be exchanged, approved and tracked. Theoretically, these steps can be managed through email, but structured, secure tools for specimen requests, tracking and MTA management can make this process easier. Specimens distributed to external collaborators for ancillary studies are often re-coded to provide greater protection of participant confidentiality and/or to blind collaborators to participant study identifiers (IDs). Maintaining links between primary study IDs and ancillary study IDs within an ASMS would provide a central location for this information and later facilitate data integration (Step 7). Also, formally tracking the samples used for ancillary analysis would facilitate planning and executing future, related studies that wish to leverage “sister” specimen vials.

When a study involves multiple labs and investigators, tracking and sharing assay results using a multiplicity of spreadsheets can quickly become unwieldy and lead to variation in formats and standards. Systems that support the definition and use of custom assay data types (*e.g.,* LabKey Server) can make it easier to collect assay data in a standard manner and share it efficiently. Such systems could be particularly helpful when assay harmonization [72,73] is needed to ensure results are comparable across labs, investigators and studies.

####  7. Data integration

After the collection of clinical data and/or execution of assays, results must be centralized and integrated to allow for comprehensive analyses. When investigators are external to the primary study, this process may be entirely external to the team (and information systems) responsible for the original study.

Receipt of results must be tracked in a manner that allows administrators to identify remaining and missing data, including the reason for missing data (*e.g.*, the subject has passed away or become unreachable). Results must also be associated with metadata indicating the processing and quality control steps that have taken place for the data, or other information necessary for understanding the context and provenance of the data. The availability of such information is becoming ever-more important for weighing the comparability of assay results thanks to the rapid evolution and growing complexity of high-throughput experimental techniques [74-78].

Results from both the original and the new study must be integrated, so tools should support the combination of data from multiple studies or protocols. Furthermore, when data representation differs between primary and ancillary studies, these differences may need to be reconciled before data can be integrated (as described in Step 5 above). The use of a single data management system for both primary and ancillary studies may reduce such divergence, for example through the enforcement of standardized terminologies and the use of standardized tools for data acquisition.

Integrated results must be available for review by a range of contributors, from principal investigators to statisticians to lab scientists who performed the assays. This means that the system for storing this information should ideally be accessible to all collaborators and allow them to contribute their updates back to the system in a manner that tracks data processing. During the entire process of data integration, the data must remain secure, so only those authorized to access particular data sets may view them.

#### 8. Data and specimen repatriation

After the ancillary study reaches completion, data produced by this study ideally needs to be contributed back to the central database for the primary study. This allows other investigators to leverage the data for further studies. Left-over specimens from the ancillary study may also need to be moved from labs, clinics, and external repositories into the central specimen repository for the primary study. This enables easier reuse of such vials in future studies. However, neither data nor specimen repatriation is straightforward, and tracking data and specimen provenance can be quite challenging.

We heard significant concerns about the difficulty of bringing ancillary data into the primary repository. One organization told us “we may never see the data [again].” Today, some organizations enforce deposition of ancillary study data into the primary study database quite strictly. They require receipt of data from the ancillary study before they release the full set of primary study data to external investigators [49,51]. For studies where full datasets from the primary study are required for the development of ancillary data, this is not practical.

Successful data repatriation requires that data, provenance notations and context information adhere to the standards and formats of the primary study. When ancillary study data have been managed in systems external to the systems employed for the primary study, this can be particularly challenging. Furthermore, information about data origins (in particular, which data came from the primary vs. the ancillary study) may no longer be available, making it hard to integrate ancillary data back into the primary study without duplicating data. When provenance information is lost and layers of an ancillary study can no longer be tracked back to their original sources, researchers run the risk of misunderstanding which data are comparable. Moreover, when the origins of clinical trial data are not clearly traceable, the trial may not meet the FDA’s ALCOA standard (attributable, legible, contemporaneous, original, and accurate) [79,80]. An ASMS that allows management of both primary and ancillary data could allow standardization of data and metadata collection throughout the study, as well as retention of data provenance information and linkages.

A further wrinkle unique to ancillary studies is that data funded and obtained by external investigators may need to be marked as embargoed for use by other investigators for some period of time. Embargo allows the data originators to produce the first publications reporting their results.

#### 9. Publication

Organizations that permit ancillary studies to be performed using their data consistently require an opportunity to review and approve manuscripts resulting from ancillary studies before the publication. This is necessary to ensure compliance with regulations (particularly participant privacy laws) and organizational policies.

Tracking of manuscripts under consideration and already published can be sufficiently complex that support from software tools is desirable. For example, to support this scenario, the HVTN utilized the Atlas Science Portal to provide a custom approval workflow for publications and other documents.

### Cross-study data pooling, reconciliation and update scenarios

The need to assemble and integrate data across studies during ancillary study workflows can pose a key impediment to using a CTMS alone for ancillary study management. As a result, cross-study challenges can prove influential in examining alternatives such as an ASMS. For this reason, we review how and when cross-study data pooling, reconciliation, and update challenges occur for ancillary studies.

The intended meaning of “cross-study data pooling” can vary widely in scope and degree of integration at different stages of ancillary study workflows and in different organizations. This variation can exert a strong influence on requirements, so recognizing this range can help an organization prioritize its informatics needs. The spectrum of cross-study data pooling scenarios varies along at least two axes:

#### Scope

• A **broad** scope of pooling is usually desirable during hypothesis generation, feasibility analysis, and proposal development and approval (Steps 1–3 of the ancillary study workflow described above and shown in Table 1). For example, to investigate exceptional responses to a disease (and inspire therapeutics), investigators may need to pool the results from many different studies to identify patterns and notable participants. Less commonly, the process of information pooling and integration (Steps 5–7) may also encompass a broad scope when results and/or specimens from a large number of primary studies are selected for reuse in an ancillary study. An information integration system such as an ASMS can be particularly helpful for broad-scope scenarios by providing a unified view across and access to a wide range of data and specimens, including external data sources.

• A **narrow** scope of pooling may be needed when the results from a limited set of studies are aggregated and integrated for reuse in an ancillary study (Steps 5–7 of the ancillary study workflow). The narrower the scope, the greater the variety of tools that can serve as viable alternatives for cross-study data pooling.

#### Degree of integration

• A **tight** degree of integration between pooled data is needed when the results of specific primary studies are aggregated and integrated with ancillary results for rigorous cross-analysis (Steps 5–7 of the ancillary study workflow). Tighter pooling may also be desirable during hypothesis generation and study planning to allow more powerful and accurate data mining (Steps 1–3). As discussed earlier (see Steps 5 and 7), tight integration of pooled data requires careful reconciliation and standardization to ensure data comparability. An ASMS can facilitate many aspects of tight data pooling, ranging from providing data reconciliation tools to pre-empting the need for reconciliation chores in the first place by facilitating good data management practices.

• **Loose** integration of pooled data may be adequate when summary reports of resource availability are sufficient for hypothesis generation, planning and study approval (Steps 1–3 of the ancillary study workflow). A unified information system like an ASMS can make it easier to automatically generate centralized, live summary roll-ups that span an organization’s data sources without manually aggregating data from different repositories. At the same time, the looser the pooling, the greater the variety of tools that may be adequate.

Actually achieving cross-study data pooling can beget further challenges in update consistency and data integrity across primary and ancillary studies. Data updates may need to flow from a primary study to a child ancillary study when data lock in the primary study occurs after ancillary study initiation. Repatriation of ancillary study data to relevant primary studies may be desirable after ancillary study completion (Step 8 in the ancillary study workflow). Repatriation can be straightforward when ancillary data are purely additive to primary data; however, such updates can be problematic when primary data have been altered in the ancillary study (*e.g.*, corrected as part of improved error detection). Moving data between studies can produce confusion over which study is the database of record for which type of data.

## Discussion

### Opportunities and challenges for ASMS use

ASMSs designed around the scenarios described here can help research organizations overcome the operational and data management challenges of ancillary studies. By providing tools for difficult, repetitive, and complex tasks, ASMSs can lower costs, increase efficiencies, produce more accurate results, expand the use of valuable primary study data, and reduce both expertise and personnel requirements. For organizations with extensive existing software systems, introducing an additional data management system may pose substantial challenges in integration, interoperability, data redundancy and data exchange. This may eventually motivate incorporation of ASMS features into CTMS systems; however, having a separate, complementary ASMS may still make sense for certain organizations.

Our collaborators exemplify users who have found value in developing ASMSs that are complementary to existing systems. Key motivators for system development have been hypothesis generation and specimen allocation for pre-clinical, not clinical, studies. Their questions have been exploratory and outside of the FDA pipeline, so they have not needed or wanted the overhead of working within a fully validated CTMS. Furthermore, their in-house software infrastructure did not provide natural points for integrating ASMS features. Some had arms-length relationships with the contract research organizations (CROs) running relevant CTMSs, while others did not employ comprehensive, integrated CTMSs. For this reason, direct interoperability of the ASMS system with a CTMS was not desired or needed.

Even when integration of ASMS features into a CTMS may be ideal, it may not be practical, so a complementary ASMS may be desirable. For an organization that already has a CTMS, updates to the CTMS system can be nontrivial, particularly when system validation is required for clinical studies. Furthermore, supporting the specimen allocation scenarios of ancillary studies requires some degree of integration with one or several LIMS, which may not be feasible for a CTMS.

Cross-study scenarios can be key motivators for adopting an ASMS, particularly when an organization already has a CTMS in place. Cross-study data pooling and integration scenarios can be problematic for a CTMS given the paucity of such support in many existing CTMSs. Furthermore, when ancillary studies need to begin before data lock of relevant primary studies, an ASMS may be needed to update and possibly reconcile ancillary study records with updates from relevant primary studies.

If an ASMS is used as a complement to existing systems, interoperability, data exchange and data integration may be somewhat eased by the further development and adoption of standards, including standardized ontologies. The arrows in Figure 1 suggest many of the potential pain points for data exchange in the workflows discussed here, including the transmission of: (1) primary study results from CTMS to ASMS, (2) specimen information from specimen repositories’ LIMS to ASMS, (3) consent information from clinical sites to ASMS or CTMS (4) specimen requests (including MTAs) from ASMS to specimen repositories, (5) specimen metadata from specimen repositories to assay labs, (6) assay results to ASMS, (7) ancillary study results from ASMS to CTMS, and (8) ancillary study results from ASMS to publications or other venues for sharing results. Additional pain points that are not shown include the transfer of (1) case report form (CRF) results to CTMS and later to ASMS; (2) primary study consent and IRB information to CTMS and later to ASMS; and (3) ancillary study consent and IRB approval to ASMS; and (4) assay results from laboratory machines to lab record systems. Data transfer becomes further complicated when upstream errors occur and changes need to propagate to downstream systems, or when quality control mistakes are identified downstream and need to propagate upstream. Even when data exchange succeeds, semantic variations in delivered data, lack of sufficient data context, and uncertain data provenance can still stymie comprehension and meaningful use.

Standards relevant to some of these data transfer and comprehension pain points are already available (*e.g.*, standards for CRF results [81]), while others are undergoing continued, active development [72,76-78,82-86]. Widely used standards have the *potential* to facilitate the reuse of study data, the automation of ancillary study workflows, and even the reuse of software systems themselves. However, in our experience developing software systems, systematic use of standards is currently more the exception than the rule. For this reason, the adoption of standards cannot be assumed as a uniform solution for many of the data transfer challenges mentioned here. This may change as NIH grant requirements drive a greater degree of adherence to standards. For example, the recent funding opportunity announcements for the competitive renewal of the HVTN [87], HPTN [88], and MTN [89] require CDISC [90] compliance for the collection, storage, and transfer of study data.

While standards do yet provide a complete solution, some degree of usage of both official standards (such as CDISC) and pervasive, industry-developed file formats can still reduce integration hurdles and foster system adoption. In our experience disseminating the LabKey Server open source platform, we have found that organizations that use data formats already incorporated in the platform (*e.g.*, Frontier Science’s LDMS [91] format for describing specimens and Clinical DataFax System Inc.’s DataFax [92] format for defining datasets and visit maps) consider this a plus for adopting LabKey Server. LabKey Server itself emphasizes well-documented archive formats [93] that convey study data via tab-separated value text files and metadata, properties, and settings via simple XML files. A variety of organizations (*e.g.*, SCHARP) use these formats to integrate external study data into their LabKey Server systems. However, we have seen few industry-defined data formats (or official, community-defined standards) that are sufficiently widely used to be significant drivers of adoption and use. Instead, providing flexibility in accommodating an organization’s existing or newly invented data types (*e.g.*, LabKey Server’s graphical wizard for describing new assay data types [30]) has proven far more important to adoption. Our experience squares with a recently published comparison of the success factors for i2b2 and REDCap (Research Electronic Data Capture) and the hindrances of caBIG (cancer Biomedical Informatics Grid) [94].

### Naming considerations

No genuinely standard terminology exists to describe systems for primary study management, let alone ancillary study management. For example, the term Clinical Data Management System (CDMS) is sometimes used as a synonym for CTMS (e.g., Ohmann *et al*[84].). Given this variability, we made a best approximation when we chose the term ASMS. In our own experience, the key use case for such a system is management of data for follow-up studies that require analysis of storied specimens. However, we define the term ASMS more broadly, encompassing both secondary data analysis studies and studies that require additional measurements (clinical, specimen-based or both). We expect an ASMS to be useful in these situations as well.

We derived the name Ancillary Study Management System based on common terminology and use cases. During our review of research network protocols, the term “ancillary studies” came up most frequently and specifically as the term for follow-up studies that encompassed more than just secondary data analysis. The appearance of the term “ancillary studies” in NIH grant announcement titles [2-9] and the frequency of this term in PubMed searches [10] (910 hits, as of December 2012) confirmed that it is in common use. We define an ASMS as a system for managing data for follow-up studies. Such studies require collection of additional measurements, usually from stored specimens, or secondary analysis of data.

### Current system development efforts

Given the calls for open source CTMSs in the recent past [19,24], we expect open source ASMSs to be desirable. In collaboration with CHAVI, the ITN and nPOD, we are currently developing a general-purpose, open-source ASMS based on the LabKey Server system. This paper does not fully explore the ancillary study features of this system because these features are currently under development.

LabKey Server is a web application implemented in Java that runs on the Apache Tomcat web server and stores its data in a relational database engine. It supports managing, integrating, analyzing and sharing a wide range of experimental and clinical data types. The system already includes many features particularly helpful for ancillary study data management, including tools for: (1) sorting, filtering, aligning, and visualizing data; (2) managing experimental data types; (3) integrating varied types of data from different sources; (4) marking columns with ontological concepts to indicate meaning and relationships; and (5) summarizing and reviewing available data in real time; and (6) performing quality control in a traceable manner. The system also already includes many features useful for operational management of ancillary studies, including: (1) role- and group-based security; (2) specimen tracking and request management (3) issue trackers; (4) wikis; (5) message boards; (6) file management tools; and (7) client libraries for building custom interfaces. The basic features and architecture of LabKey Server are explored elsewhere [30,32,33].

Current and future development efforts address five key ancillary study scenarios: (1) sub-setting, tracking, and pooling participants within studies; (2) reporting and analysis at the participant group level; (3) scheduling and tracking expectations; (4) providing data context; and (5) tracing data provenance. The current LabKey Server release (v12.3) already provides support for notable portions of the first four of these scenarios and a few cases of the fifth.

Source code, documentation, tutorials and videos for LabKey Server are freely available under the Apache 2.0 license [95] from the LabKey Software Foundation at http://www.labkey.org. Stable, installable releases of the system are freely available from LabKey Software at http://www.labkey.com. LabKey Server is maintained by a team of professional software engineers.

### ASMS design strategies for addressing cross-study challenges

A well-designed, robust ASMS can help overcome cross-study challenges, both by providing tools and by facilitating best practices. As mentioned previously, cross-study scenarios (particularly data aggregation, reconciliation, integration, and revision) can prove particularly influential in the decision to adopt an ASMS. To demonstrate the feasibility of using an ASMS to address such issues, we highlight a few of the ASMS design strategies we have used in developing LabKey Server’s ASMS features and in advising organizations on best practices:

1. **Emphasize and support data management strategies that produce standardized, comparable data.** The cleanest way to deal with data reconciliation challenges is simply to pre-empt them. This requires a multifaceted approach, including good data management practices, collaborative planning, long-term thinking and robust information systems. For example, establishing common, uniform vocabularies and ontologies can aid standardization; however, enforcement may require providing informatics tools that support vocabulary specification for data entry.

2. **Recognize that human review and judgment are essential, so make them easier.** Automated methods for combining data may be convenient, but they are not always justifiable. Human judgment must still play a role in ensuring that data are comparable and/or in making data comparable. To facilitate human judgment, it is helpful to provide context and provenance information alongside data, as well as to assist the tracking and collection of this type of information. For example, LabKey Server helps users associate protocols with studies, enter structured metadata alongside experimental data, associate transformation scripts with imported data, and tag excluded data with color-coded quality control flags.

3. **Provide tools and strategies for standardizing and reconciling data in bulk, particularly during import.** Standardizing data during the import process itself makes it easier to track how and when the data were transformed, plus avoid identifier conflicts that would (by design) prevent data import. LabKey Server supports visit mapping, so visits of the same type or range of dates can be aliased to the same canonical visit identifiers. To prevent conflicts between participant identifiers, our collaborators often prefix a study identifier before each one to ensure uniqueness during import to LabKey Server. To standardize column names, users can specify column metadata “import aliases” that map to canonical column headers. Various scripting options are available for field-by-field transformations. For example, the system supports the association of a sequence of scripts with a versioned pipeline that reproducibly transforms experimental data during import. To aid verification of expected values, column metadata can specify validators (*e.g.*, regular expressions, expected ranges, and type constraints) that are applied during data import, plus trigger color coding of invalid or missing data. Other quality control mechanisms (such as missing value indicators and quality control states) can further aid data reconciliation and standardization.

4. **Provide access to external data without requiring that it be brought into the ASMS.** External data sources (*e.g.*, a CTMS database or a SAS repository) can be made directly accessible within a LabKey Server. This can provide visibility across all available datasets and facilitate incorporation of external data into ancillary studies. Access to external data is dynamic, meaning that any modifications to such data within an external repository are immediately viewable on the associated LabKey Server.

5. **Provide tools for looking across all available data, both built-in and custom.** LabKey Server’s query tools support drawing in data from multiple studies and placing filtered versions of this data into ancillary studies, as well as displaying summary views. A variety of common summary dashboards are already built into LabKey Server. The system also provides full-text search for many types of data and documents, plus "study-aware" search for relevant concepts, particularly participant identifiers and study properties.

6. **Support “live updates” of primary study data contained within an ancillary study.** When an ancillary study is initiated before a primary study is complete, it may be desirable for changes to the primary study (including specimen information, such as location and available vials) to flow automatically into equivalent datasets in the ancillary study. However, such automatic updates can make it hard to know which versions of primary data have been used in derived materials (such as preliminary figures). To ameliorate this versioning challenge, a snapshot of the ancillary study itself may be taken, or the derived materials may themselves be displayed live on the server (both options LabKey Server supports). LabKey Server implements the “live update” option by requiring that primary study datasets exist separately from ancillary datasets. This assumption works well for “freezer studies,” where no new participant visits occur that might cause extension of primary datasets.

7. **Support creating ancillary studies based on “snapshot” subsets of primary study data.** This option minimizes confusion about which version of primary study data the ancillary study contains. Capturing and archiving a study snapshot at a particular point in time can also provide traceability and reproducibility when researchers publish on intermediate results before a study has completed and locked.

## Conclusion

The scenarios and requirements described here can help guide the development of systems that can make conducting ancillary studies easier, less expensive and less error prone. Given the relatively consistent characteristics and challenges of ancillary study management, general-purpose ASMSs are likely to be useful to a wide range of organizations. Open source, general-purpose ASMSs (such as the one we are developing as part of LabKey Server) are particularly desirable due to their lack of licensing fees and openness to customization by individual organizations.

## Abbreviations

AIDS: Acquired immune deficiency syndrome; ALCOA: Attributable, legible, contemporaneous, original, and accurate; ASMS: Ancillary study management system; BSI: Biological Specimen Inventory System; caBIG: Cancer Biomedical Informatics Grid; CAVD: The Collaboration for AIDS Vaccine Discovery; CDISC: Clinical Data Interchange Standards Consortium; CHAVI: The Center for HIV Vaccine Immunology; CDMS: Clinical data management system; CRF: Case report form; CRO: Clinical research organization; CTMS: Clinical trial management system; FDA: Federal Drug Administration; FTP: File transfer protocol; GCDMP: Good clinical data management practice; HIV: Human immunodeficiency virus; HPTN: the HIV Prevention Trials Network; HVTN: The HIV Vaccine Trials Network; i2b2: Informatics for Integrating Biology and the Bedside; ID: Identifier; IRB: Institutional review board; ITN: The Immune Tolerance Network; JDRF: Juvenile Diabetes Research Foundation; LDMS: Laboratory Data Management System; LIMS: Laboratory information management system; MHRP: The U.S. Military HIV Research Program; MTA: Material transfer agreement; MTN: The Mircrobicide Trials Network; NIAID: National Institute of Allergy and Infectious Diseases; NIH: National Institute of Health; nPOD: Network for Pancreatic Organ Donors with Diabetes; REDCap: Research Electronic Data Capture; RV144: The Thai Phase III HIV vaccine clinical trial; SCHARP: The Statistical Center for HIV/AIDS Research & Prevention at the Fred Hutchinson Cancer Research Center; SPORE: Specialized program of research excellence; STEP: A phase II HIV vaccine clinical trial; VISC: Vaccine Immunology Statistical Center; WHI: The Women’s Health Initiative; XML: Extensible markup language.

## Competing interests

EKN, BP, AR, and MI are employees of LabKey Software, a software consulting company that provides development, customization, and support for LabKey Server. LabKey Server is open source and freely available, so these authors do not have a direct financial interest in the software itself.

## Authors’ contributions

All authors contributed ideas and edits to this manuscript. MI initially suggested the paper topic. EKN researched and wrote the paper. BP, AR, SR, DH, SA, AA, and MI are involved in the design of LabKey Server’s support for ancillary study data management scenarios. BP, AR, MI, and EKN are involved in the development of these features.

## Pre-publication history

The pre-publication history for this paper can be accessed here:

http://www.biomedcentral.com/1472-6947/13/5/prepub
